# Supplementary material for: Reconciling Mining with the Conservation of Cave Biodiversity: A Quantitative Baseline to Help Establish Conservation Priorities
Source: PLoS One. 2016 Dec 20;11(12):e0168348. doi: 10.1371/journal.pone.0168348 (PMC5173368; doi:10.1371/journal.pone.0168348)
Supplement: S1 Dataset — (ZIP) [file pone.0168348.s002.zip › Taxa/Serra Norte/SN_2013/N4WS/Lista N4WS-31.pdf]

CAVIDADE N4WS-0031

Classe	Ordem	Fam/Outros	Gên/Outros	Espécie	S-U	U-S	Zon.
Arachnida	Acari	Laelapidae		sp.3	X		P
Arachnida	Amblypygi	Charinidae	*Charinus*	sp.1	1		P
Arachnida	Amblypygi	Phrynidae	*Heterophrynus*	*longicornis*	2	2	P
Arachnida	Araneae	Ctenidae		jovem	1		P
Arachnida	Araneae	Ochyroceratidae	*Ochyrocera*	sp.1		X	P
Arachnida	Araneae	Pholcidae	*Leptopholcus*	sp.1		X	P
Arachnida	Araneae	Pholcidae	*Mesabolivar*	*eberhard*	X		P
Arachnida	Araneae	Theridiosomatidae	*Plato*	sp.1	X	X	P
Arachnida	Pseudoscorpiones	Chthoniidae	*Pseudochthonius*	sp.1		X	P
Entognatha	Collembola	Cyphoderidae		sp.1	X		P
Entognatha	Collembola	Paronellidae		sp.4		X	P
Insecta	Coleoptera	Curculionidae	Scolytinae	sp.6	X		E
Insecta	Coleoptera	Staphylinidae		jovem	X		E
Insecta	Diptera	Cecidomyiidae	Cecidomyiinae	sp.		X	P
Insecta	Diptera	Ceratopogonidae		sp.	X		P
Insecta	Diptera	Drosophilidae	*Drosophila*	*eleonorae*	X		P
Insecta	Diptera	Phoridae	Metopininae	sp.	X		P
Insecta	Diptera	Phoridae	Phorinae	sp.	X		P
Insecta	Diptera	Psychodidae	*Sciopemyia*	*sordellii*	X	X	P
Insecta	Heteroptera	Reduviidae	Emesinae	sp.1		1	P
Insecta	Homoptera	Cixiidae		sp.1		X	P
Insecta	Homoptera	Cixiidae		sp.3	X		E
Insecta	Hymenoptera	Formicidae	*Camponotus*	*atriceps*		2	P
Insecta	Hymenoptera	Formicidae	*Camponotus*	sp.1	X		P
Insecta	Hymenoptera	Formicidae	*Neivamyrmex*	sp.1	X		P
Insecta	Isoptera	Termitidae	*Nasutitermes*	sp.	X	X	P
Insecta	Lepidoptera	Noctuoidea		sp.1	X		P
Insecta	Lepidoptera	Noctuoidea		sp.2		1	P
Insecta	Orthoptera	Phalangopsidae	*Paraclodes*	sp.	11		E
Insecta	Orthoptera	Phalangopsidae	*Phalangopsis*	sp.	1	4	P
Gastropoda	Pulmonata	Systrophiidae	*Happia*	sp.	X		P
Amphibia	Anura	Strabomantidae	*Pristimantis*	cf. *fenestratus*		1	E
Mammalia	Chiroptera	Emballonuridae	*Peropteryx*	sp.	1		P
